# Validating gut flushing as a non‐lethal sampling technique for diet monitoring in agastric fish

**DOI:** 10.1111/jfb.70301

**Published:** 2025-12-22

**Authors:** Lenka Kajgrova, Petr Blabolil, Vladislav Draštík, Luboš Kočvara, Tomáš Jůza, Martin Bláha

**Affiliations:** ^1^ Biology Centre CAS Institute of Hydrobiology Ceske Budejovice Czech Republic; ^2^ University of South Bohemia in Ceske Budejovice Faculty of Fisheries and Protection of Waters Vodňany Czech Republic; ^3^ University of South Bohemia in Ceske Budejovice Faculty of Science Ceske Budejovice Czech Republic

**Keywords:** common carp, dietary studies, fish welfare, gut content analysis, long‐term monitoring

## Abstract

Non‐lethal diet sampling is essential where individual‐level monitoring and welfare matter, yet evidence on season‐long outcomes in agastric fishes is scarce. We tested syringe‐based gut flushing in pond‐reared common carp *Cyprinus carpio* over one growing season (April–September) using PIT‐tagged fish assigned a priori to three handling histories: flushed (sedated, measured, flushed), handled‐only (sedated, measured), and unhandled controls. At harvest, recapture (survival proxy) remained high and statistically similar among handled groups and growth to harvest did not differ across treatments (end‐point mass: flushed 1857.3 ± 367.4 g; handled‐only 1769.7 ± 311.8 g; unhandled 1731.9 ± 392.6 g). A mid‐season divergence in July (handled‐only > flushed in weight gain) was transient and absent at harvest. Operational performance scaled predictably: total water required to clear effluent increased with fish size (~20 mL in small carp to >90 mL in large individuals; occasional ~120 mL) and varied seasonally, rising from May to August and easing in September. Practical safeguards (rounded catheters guided between pharyngeal teeth; conservative advancement ≤2 cm beyond the teeth; controlled bolus infusions; standardised recovery) supported complete evacuations with low injury risk. Collectively, these results validate gut flushing as a field‐ready, non‐lethal method that can be integrated into routine dietary monitoring of agastric cyprinids without compromising survival or harvest growth, while providing simple, size‐ and season‐based parameters to standardise protocols across studies.

## 1 INTRODUCTION

Most of our current knowledge on the trophic ecology and feeding interactions of fish comes from dietary studies based on gut content analysis, providing valuable insights into fish biology and their roles in aquatic ecosystems (Amundsen & Sánchez‐Hernández, 2019; Hynes, 1950; Hyslop, 1980; Manko, 2016; Seaburg, 1957; Šusta, 1888). Dietary studies are important for understanding dynamics in natural habitats as well as informing aquaculture practices, where knowledge of feeding behaviour helps optimise the growth and feed efficiency of cultured fish (e.g. Manko, 2016; Roy et al., 2022). Although a variety of methodologies are employed to investigate fish‐feeding ecology, each has inherent constraints regarding taxonomic resolution, feasibility and ethical considerations (e.g. Manko, 2016; Vejřík et al., 2024). The most established method used in the study of the feeding habits of fish and other animals is based on stomach or gut content analysis that allows for the identification of ingested prey and provides detailed, short‐term dietary data (Buckland et al., 2017; Zacharia & Abdurahiman, 2004). With the onset of modern methods of stable isotope or fatty acid analysis, direct observations or metabarcoding, one can obtain information on trophic positioning within food webs or foraging behaviour and even a variety of species‐specific prey items that might be overlooked in direct observation. As a trade‐off, these methods typically require more complex workflows and higher costs (Braga et al., 2012; Kihlberg et al., 2023; Vejřík et al., 2024). On the other hand, direct methods provide fine taxonomic detail but might be biased by the prey conditions or differential digestion of sampled fish species (Buckland et al., 2017; Calver & Loneragan, 2024).

Despite modern methods, gut content analysis remains the primary tool for direct dietary assessment, which brings more valuable information in combination with modern approaches (e.g. Davis et al., 2012; Palacios‐Narváez et al., 2024; Rybczynski et al., 2008; Vašek et al., 2018). The collection of gut or stomach content samples includes two techniques. The first is lethal and involves dissection of the stomach/gut after the fish has been killed, and the other is non‐lethal, involving flushing, lavage and other approaches (for detailed methods and their modification, see Kamler & Pope, 2001). Although both techniques provide direct evidence of dietary composition, prey selection and feeding strategies, lethal methods provide more accurate dietary data by ensuring complete retrieval of all gut contents. However, this method limits the ability to conduct repeated sampling on the individual level and long‐term dietary monitoring of individuals (Manko, 2016). Moreover, this method might be inconvenient for studying endangered, economically valuable fish and/or those from low‐density populations (Chipps & Garvey, 2007). Because of this, stomach flushing, or gastric lavage, has been widely used for gut content retrieval in most fish (Hyslop, 1980). The technique involves using pressurised water to expel gut contents through the mouth or anus, depending on whether the fish possesses a true stomach, typically as one or more discrete bolus infusions administered within a single treatment event until the effluent runs clear (Manko, 2016). The method has been refined ever since its first application, with improvements in catheter design, water pressure regulation and fish handling procedures to minimise stress and injury (Chipps & Garvey, 2007; Hyslop, 1980; Kamler & Pope, 2001; Manko, 2016).

Despite the broad adoption of non‐lethal techniques, most studies have focused on retrieval success and the short‐term impact on fish survival, leaving a gap in our understanding of how these methods affect fish physiology over time (for review, see, e.g., Kamler & Pope, 2001). The data lack details on the effects of syringe‐based flushing (delivered as discrete bolus infusions within a single treatment) and associated handling on growth and survival over a growing season. Moreover, standard flushing protocols often require adjustment depending on anatomical differences, such as the lack of a true stomach in agastric fishes (Manko, 2016). This study aimed to evaluate the safety and applicability of a syringe‐based gut‐flushing technique in the common carp *Cyprinus carpio* Linnaeus, 1758 (hereafter carp), a model agastric species commonly used in aquaculture. Specifically, we (i) compared survival and growth across three handling histories—flushed, handled‐only (sedated and measured, no flushing) and unhandled controls—over a full growing season in semi‐intensive ponds, and (ii) quantified how fish size and season influenced the water volume required to achieve clear‐effluent evacuation. By reporting size–season relationships for flushing volumes and clarifying handling safeguards, our study provides practical insight into using this non‐lethal method in the dietary studies of cyprinids and other agastric freshwater fish.

## 2 METHODS

### 2.1 Study species and experimental conditions

This study used carp, a model agastric fish with a simple S‐shaped gut. Fish were reared over one growing season (from April to September 2023) in three similar ponds (herein referred to as ponds A, B and C, each 0.16 ha, mean depth 0.8 m) following the common practice of semi‐intensive fish farming (see Kajgrová et al., 2024). The ponds were located next to each other at the experimental pond facility of the University of South Bohemia in Vodňany, Czechia (Central Europe, GPS 49°09′24.9″N, 14°09′54.2″E, 404 m a.s.l.) and had a single inflow from the Blanice River (Figure S1). Ponds were operated identically (no feed beyond standard facility practice, identical predator stocking).

In‐situ physical–chemical parameters, namely water temperature, pH, oxygen saturation and conductivity, were measured in situ 20 cm below the surface using a multimeter (HI98494; Hanna Instruments) and remained within physiological ranges suitable for carp (Table 1 and Figure S2). Water transparency was assessed using a Secchi disk. Field measurements coincided with fish sampling dates.

**TABLE 1:** Environmental conditions in three experimental ponds during seven monthly sampling campaigns from April to October.

Pond	A	B	C
Water temperature (°C)	16.7 (9.7–24.5)	16.8 (9.9–24.3)	17.5 (9.8–23.9)
Oxygen saturation (%)	81 (60–172)	81 (46–171)	83 (53–177)
Transparency (cm)	33 (15–110)	33 (15–100)	38 (20–110)
Conductivity (μS cm^−1^)	140 (125–192)	152 (123–207)	138 (121–203)
pH	8.7 (7.1–9.3)	8.0 (6.1–9.12)	7.9 (7.0–9.1)

*Note*: Values are presented as the median with the minimum and maximum range (median, min–max). The seasonal trends are shown in Figure S2.

Each pond was stocked on 3 April 2023 with 210 carp of the same size class (age 2+, total length [TL] 282 ± 18 mm, body weight [BW] 367 ± 69 g, mean ± SD), totalling 77 kg per pond. All fish received subdermal passive integrated transponder (PIT) tags (Biomark GPT12; Biomark Inc.) at stocking. Tags were scanned with a handheld RFID reader (Datamars TracKing‐1 EID) at stocking, during monthly sampling of a subset (10 May, 8 June, 12 July, 9 August, 6 September 2023) and at final harvest (16 October 2023). This enabled individual‐level tracking and comparisons of growth and survival across handling treatments. At harvest, all fish were scanned, counted and measured (TL to 1 mm, BW to 1 g). To suppress unwanted fish that could affect carp welfare, each pond received the same predator stocking of pikeperch *Sander lucioperca* (Linnaeus, 1758) (45 fish, TL 207 ± 47 mm; for more details see Jůza et al., 2024), following standard practice, with identical predator numbers across ponds to avoid between‐pond bias.

The care and use of experimental animals complied with the EU harmonised Animal Welfare Act of the Czech Republic. The research unit is licensed (No. 53100/2013‐MZE‐17214) under the Czech National Directive (the Law against Animal Cruelty, No. 246/1992). All procedures were performed under this licence and followed internal standard operating procedures for sedation, handling and recovery.

### 2.2 Experimental design and fish handling

Monthly fish sampling (May to September 2023) was performed by seine‐netting (mesh size 4 cm, length 20 m, height 2 m). In each sampling campaign, 20–25 randomly selected fish were measured for individual TL and BW (to 1 mm and 1 g, respectively); from these, six randomly chosen fish were selected for the gut‐flushing technique. At each sampling campaign, the captured fish were scanned for PIT tags. Sampling in April was not conducted to allow the fish to acclimate to the pond environment and to minimise handling stress. Similarly, further to limit stress, sampling was omitted in October when the fish were harvested.

For analyses, fish were classified a priori into three handling groups throughout the study:

- Flushed: monthly sampled carp that, after sedation and biometrics, underwent gut flushing followed by recovery.
- Handled‐only: monthly sampled carp that underwent the identical workflow (sedation, biometrics and KMnO₄ recovery) but without gut flushing.
- Unhandled controls: carp not handled between stocking and harvest; PIT‐tagged and stocked at baseline and encountered again only at harvest.

### 2.3 Sedation and recovery

Sedation and recovery were applied to the flushed and handled‐only groups. Selected fish were sedated before any procedure to minimise stress and handling‐related injuries. A 2‐phenoxyethanol solution (0.3–0.4 mL L^−1^; Tsantilas et al., 2006) was prepared in an aerated water bath. Fish were individually transferred to the bath for sedation. The sedation process was carefully monitored, and the fish were removed once opercular movement visibly slowed, corresponding to stage 3a–3b anaesthesia (loss of righting reflex with slow opercular rate). All handling surfaces were pre‐wetted. Recovery was in aerated water with KMnO₄ 1 mg L^−1^ with checks at approximately 1, 6, 12 and 24 h.

### 2.4 Gut flushing procedure

Gut flushing was performed on only the flushed group. The gut flushing procedure involved the use of a Janett 150‐mL syringe (Alfa Vita) attached to a flexible polyethene catheter, with the external diameter selected based on fish size (Figure S3). The catheter tips were rounded (heat/sanding) to avoid mucosal injury. For smaller carp (<400 g), soft catheters of 3–5 mm in diameter (usually supplied with the Janett syringe) were sufficient (Figure S3). Stiffer catheters were preferred for larger fish (>400 g) to facilitate insertion past the pharyngeal teeth. Catheter diameter was adjusted accordingly: fish weighing 300–500 g received a 6‐mm catheter, those between 500 and 800 g were flushed using a 7.6‐mm catheter and fish over 800 g required a 9‐mm catheter. The heaviest fish flushed in this study weighed 2.2 kg, and for such individuals, catheters made from more rigid materials were advantageous to withstand pressure from the pharyngeal teeth. Based on practical experience, in some cases, smaller‐diameter catheters than expected for a given weight category were used to ease insertion. Before insertion, the catheter was lubricated with a sterile saline solution to reduce friction and minimise injury risk.

**FIGURE 1:**
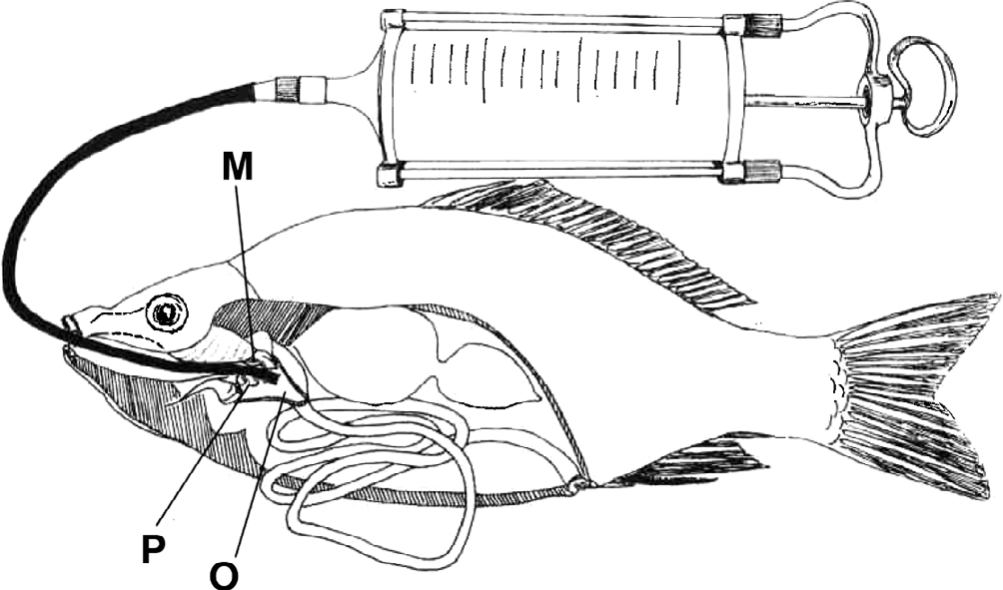
The original scheme of Faina (1983) shows the positioning of the catheter between pharyngeal teeth (P) and the masticatory plate of the os basioccipitalis (M) in the oesophagus (O).

The gut flushing procedure involved securely holding the fish in a dorsal position to keep the mouth open for catheter insertion (Figure S4). The catheter was gently inserted through the oral cavity and advanced approximately 3–8 cm from the mouth opening into the oesophagus, with insertion of the catheter between pharyngeal teeth and the masticatory plate of the os basioccipitalis (Figure 1). Lukewarm, dechlorinated water (18–22°C) was delivered as discrete bolus infusions at approximately 5–10 mL s^−1^, with water pressure carefully regulated to minimise stress. Expelled gut contents were collected in a pre‐labelled container, and the process was repeated in approximately 10‐mL increments until the expelled water ran clear, indicating complete gut evacuation. Mean total volume was approximately 39 mL and never exceeded syringe capacity. The catheter was then carefully withdrawn. If intestinal filling occurred without effluent, infusion was aborded to avoid overpressure and potential fish injury; such fish were immediately transferred to the recovery bath and logged as aborted.

Following gut flushing, fish were placed into a separate aerated recovery tank containing potassium permanganate (KMnO₄) at 1 mg L^−1^ to aid in mucosal healing. Fish were observed for immediate signs of distress, including abnormal buoyancy or erratic swimming. Post‐procedure mortality was monitored at 1, 6, 12 and 24 h. Monthly sampled carp assigned to the handled‐only group underwent the same procedures—sedation, biometric measurements (TL, BW) and placement in KMnO₄—except for the flushing process. Fish in group unhandled were not handled during the growing season; they were tagged, measured and stocked at the start, and measured only at harvest.

### 2.5 Statistical analyses

Analyses focused on the effect of handling treatment with three a priori levels (flushed, handled‐only, unhandled controls), with pond included as a random factor where specified. Survival was calculated as the proportion of recaptured fish at the final harvest relative to the number of stocked fish in each pond, and the three treatments were compared using a one‐way analysis of variance (ANOVA). Survival between flushed and handled‐only carp in individual months was tested by linear mixed‐effects models (LMEs) with treatment as a fixed effect and pond identity as a random factor.

The carp growth was evaluated as the ratio of TL and BW at final harvest relative to TL and BW at stocking between flushed and handled‐only fish; unhandled controls were not measured mid‐season and were included in harvest‐level growth comparisons only. Growth performance (separately for TL and BW) was tested by LME with treatment (flushed, handled‐only and unhandled) as the fixed effect and the pond as a random factor to account for environmental variability among study sites, fitting (i) growth ~ treatment + (1 | pond identity) and (ii) growth ~1 + (1 | pond identity) and comparison with *anova* function. Analyses were performed in R (R Core Team, 2023) using the package lme4 (Bates et al., 2015).

Within the flushed group, the relationship between biometric parameters (TL, BW) and flushing water volume was evaluated by regression (volume vs. TL, volume vs. BW). Water volumes used for carp flushing in individual samplings were compared by ANOVA followed by a pairwise Tukey honestly significant difference (HSD) test.

## 3 RESULTS

Environmental conditions were similar among ponds. Water temperature was unimodal, peaking in July (average 25°C), oxygen saturation and transparency declined slightly through the season (averages 92% and 58 cm, respectively), conductivity and pH were relatively stable (averages 149 μS cm^−1^ and 8.1, respectively) (Figure S2). Trends were consistent across ponds with no significant between‐pond differences (ANOVA, all *p* > 0.05).

Survival did not differ between flushed and handled‐only fish (*χ* ^2^ = 0.01, *df* = 1, *p* = 0.942). In total, 74/80 gut‐flushed carp were recaptured at harvest (92.5%), 95/105 handled‐only (measured without flushing) were recaptured (90.5%) and 361/445 unhandled carp were recaptured (81.1%). Growth to harvest did not differ among the three handling groups (TL: *χ* ^2^ = 2.37, *df* = 2, *p* = 0.305; BW: *χ* ^2^ = 1.83, *df* = 2, *p* = 0.399). At harvest, mean body weight was 1857.3 ± 367.4 g in flushed fish, 1769.7 ± 311.8 g in handled‐only fish and 1731.9 ± 392.6 g in unhandled controls, consistent with the non‐significant overall treatment effect on growth.

**FIGURE 2:**
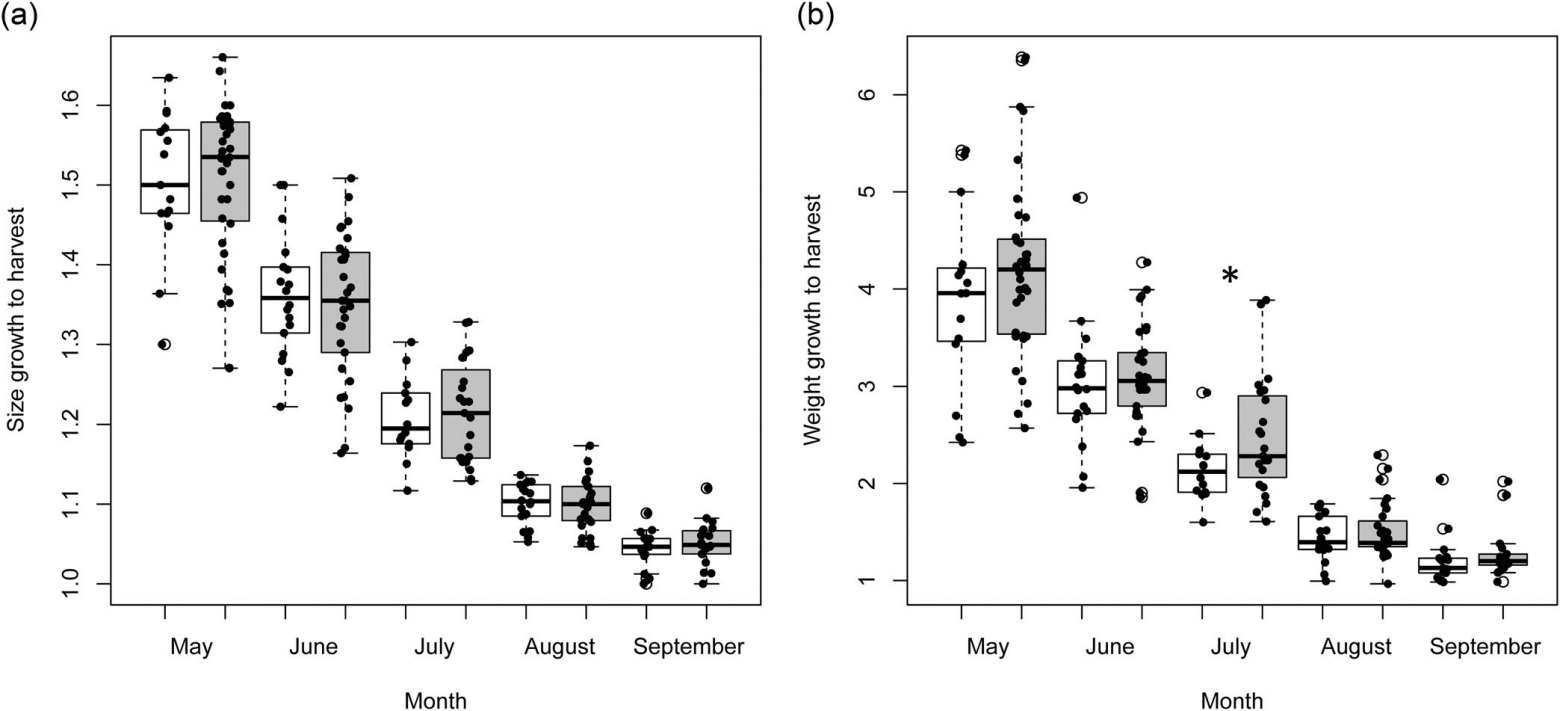
Box and whiskers plots (median, bold line; quartiles, box; minimum and maximum, whiskers and outliers, empty circles) of (a) size growth to harvest and (b) weight growth to harvest ratio, with white indicating flushed fish and grey representing handled‐only fish (not flushed, but handled). The asterisk in Figure B indicates significant differences. Jittered points indicate individual observations (*n* = 219).

Month‐to‐harvest tests showed no differences in total length between flushed and handled‐only fish (all *p* > 0.05; Figure 2a). For weight, groups were generally similar except in July, when handled‐only fish gained more than flushed fish (*χ* ^2^ = 4.29, *df* = 1, *p* = 0.038; Figure 2b).

**FIGURE 3:**
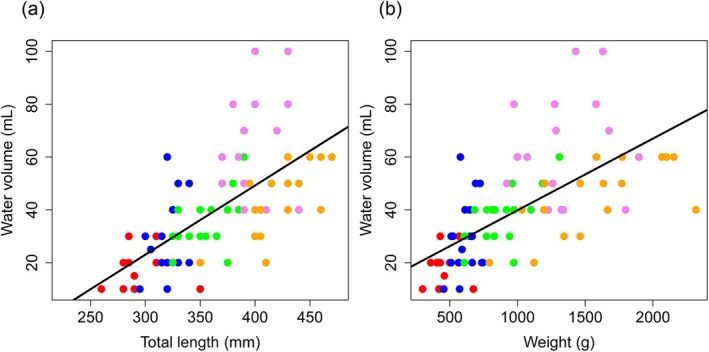
Relationship between the water volume used for flushing and (a) total length (*y* = 0.262*x*–55.586) and (b) body weight (*y* = 0.027*x* + 12.65) of fish. Each point represents an individual measurement (*n* = 86), with different colours indicating sampling events: red, May; blue, June; green, July; violet, August; orange, September.

**FIGURE 4:**
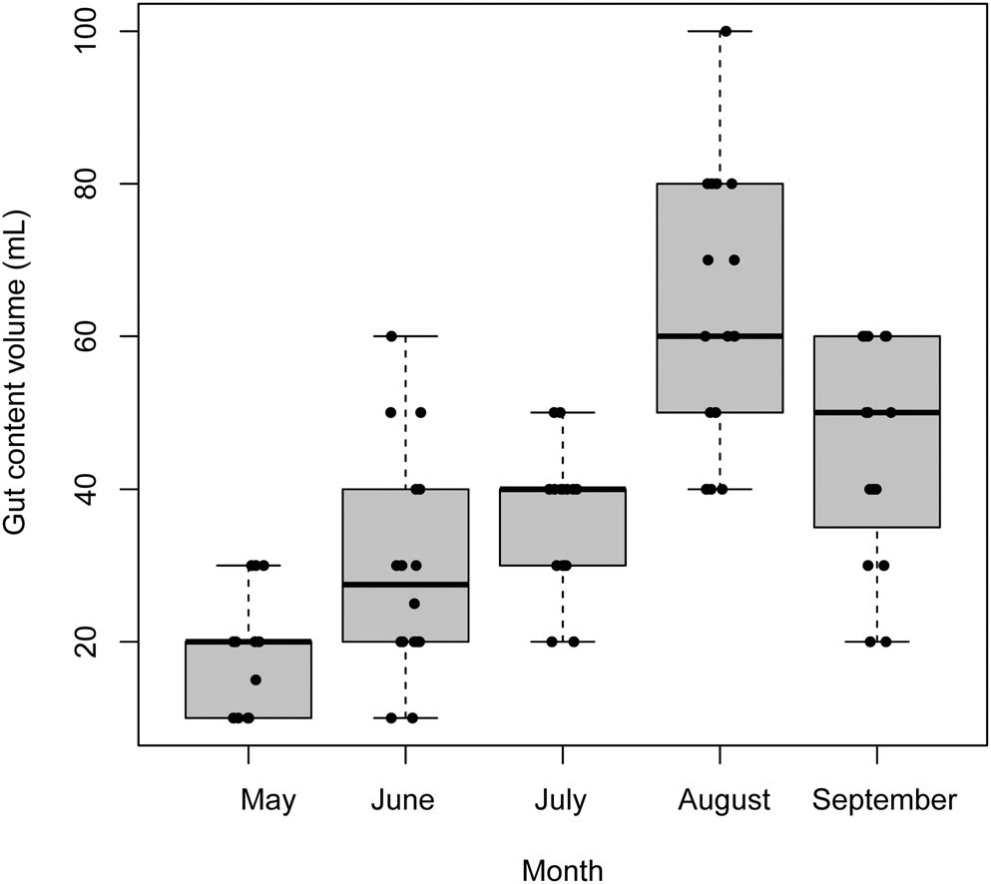
Box and whiskers plots (median, bold line; quartiles, grey box; minimum and maximum, whiskers) of seasonal variation in the volume of water used for flushing (mL) across sampling months. The letters above box plots indicate significant differences between groups based on the Tukey honestly significant difference (HSD) test. Jittered points indicate individual observations (*n* = 83).

The water volume required for complete gut evacuation increased with fish size: ~20 mL for small individuals (<350 g) and >90 mL for larger fish (>800 g), with some individuals requiring up to 120 mL. Regression confirmed positive relationships between flushing volume and TL (*F*_1,84_ = 70.0, *p* < 0.001, *R* ^2^ = 0.45; Figure 3a) and between volume and BW (*F*_1,84_ = 61.6, *p* < 0.001, *R* ^2^ = 0.42; Figure 3b). Required volume also varied seasonally (ANOVA, *F*_4,78_ = 27.2, *p* < 0.001), increasing from May to August, peaking in late summer and declining slightly in September (Tukey HSD; Figure 4).

## 4 DISCUSSION

### 4.1 Survival and growth

Our study demonstrated that the gut‐flushing procedure had no significant effect on the survival and growth of the carp, a model agastric species, over a 6‐month period in a controlled outdoor setting. The survival of flushed individuals accounted for 92.5% at harvest, which was comparable to or exceeded the survival of non‐flushed fish. In pooled terms, survival remained high across all treatments (survivability in flushed and handled‐only both high compared to unhandled), and end‐point body mass at harvest aligned with this pattern (flushed ≥ handled‐only ≥ unhandled), indicating no sustained growth penalty attributable to flushing. Our findings are consistent with previous studies reporting survival rates ranging from 60% to 100%, depending on species and handling protocol (e.g. Giles, 1980; Hakala & Johnson, 2004; Hartleb & Moring, 1995; Light et al., 1983).

While variations in growth were detected in July, the direction of the effect was transient and favoured the handled‐only fish (greater mid‐season weight gain) relative to flushed fish, with trajectories converging by harvest. This pattern is consistent with a short‐lived handling‐history effect rather than a durable cost of flushing, and parallels observations in gastric species where impacts are confined to the immediate post‐treatment window (e.g. Hakala & Johnson, 2004). Moreover, the July divergence coincided with peak temperatures, reduced transparency and lower oxygen (a pattern typical of traditional production ponds; Kajgrová et al., 2024), which might alter and/or reflect prey quality (Roy et al., 2022, 2024) and amplify small differences arising from recent handling. Between‐pond confounding is unlikely: ponds were similar in morphometry, environmental variables followed comparable seasonal trends and did not differ among ponds, predator stocking was identical and no undesirable fish were detected at harvest. Because such fishes can depress carp performance via food competition (e.g. invasive topmouth gudgeon *Pseudorasbora parva* [Temminck & Schlegel, 1846]; Kajgrová et al., 2022), their absence, together with stable environmental ranges, likely limited external pressures and supported the realignment of growth by season's end. All in all, relatively stable environmental conditions, post‐flushing recovery baths and minimised handling time in our study likely minimised further external stressors that might impact fish physiology (Portz et al., 2006; Sloman et al., 2019).

Our study involved flushing over an entire growing season, yet no cumulative negative effects on survival or growth were observed. These results are consistent with those of Giles (1980), who repeatedly sampled European perch *Perca fluviatilis* Linnaeus, 1758 using flushing methods and observed high survival and normal feeding activity after multiple recaptures. Although many existing studies (e.g. Hakala & Johnson, 2004; Meehan & Miller, 1978) only tested survivability within a few days post‐flushing, they also found no negative effect on growth and condition in flushed fish. Additionally, the trajectory of fish growth in our experiment did not deviate from patterns commonly observed in pond‐reared carp (e.g. Roy et al., 2023; Stanivuk et al., 2024).

### 4.2 Size and season variations

We observed that the volume of water required for effective flushing varied both seasonally and with fish size. The volume of water required for complete gut evacuation increased over the season, peaking in August and slightly declining in September. This pattern likely reflects higher metabolic activity and feeding intensity in warmer months (Roy et al., 2022, 2024). Moreover, gut content retrieval is influenced by fish size and anatomy (e.g. Hakala & Johnson, 2004; Hartleb & Moring, 1995; Light et al., 1983). Operationally, a simple rule‐set emerged: larger carp and late‐summer samples required greater volumes, smaller‐than‐expected catheter diameters often improved guidance between the pharyngeal teeth, and rounded, lubricated tips reduced insertion forces—all of which favoured complete yet low‐injury evacuations. This aligns with current recommendations that device dimensions and hydraulic force should be scaled to species' anatomy and size (Amundsen & Sánchez‐Hernández, 2019).

Unlike fish species with a well‐developed stomach, in which prey retention during flushing may occur (e.g. largemouth bass; Hakala & Johnson, 2004), the vast majority of fish in our study appeared to have their guts fully evacuated after the procedure. This is likely due to the anatomical simplicity of agastric cyprinids, which possess a continuous intestinal tract without a pyloric sphincter or compartmentalised stomach regions (Faina, 1983; Manko, 2016). Flushing was repeated until only clear water came out, signalling a complete gut evacuation (Faina, 1983). We noted, however, that in rare cases (fewer than five fish in total), we aborted the procedure per our a priori stopping rules when coarse or bulky feed items (e.g. whole wheat grains) likely impeded safe water passage through the intestinal tract. These events were infrequent and distributed across months when wheat was fed. Indeed, as with any non‐lethal sampling, the effectiveness in retrieving all gut content depends on species‐specific gut morphology, fish size, chosen technique and perhaps fish diet (see Kamler & Pope, 2001). In our case, the combination of anatomical characteristics of carp, the use of a controlled syringe‐based flushing technique and the adjustment of catheter size according to fish size yielded consistently high flushing efficiency across individuals, with only the rare welfare‐driven aborts noted above.

### 4.3 Methodological considerations and practical applications

Researchers have a variety of non‐lethal methods for retrieving fish stomach contents, ranging from emetics and suction devices to mechanical flushing systems (Kamler & Pope, 2001; Manko, 2016). The choice of method depends largely on the species' digestive anatomy, size range and field conditions. Our study applied a modified syringe‐based flushing technique tailored to agastric fish, as previously described in Faina (1983). The method's strengths are notably portability, power independence and low cost—features that matter when sampling must be repeated through the season and infrastructure is limited. It requires minimal equipment and is easy to implement under pond aquaculture or remote monitoring conditions. The technique is adaptable across a wide range of fish sizes, as shown by the successful flushing of carp from subadult stages up to 2.2 kg (Faina, 1983, this study). This flexibility is achieved by adjusting both catheter size and water volume to the individual fish's body size.

The choice of catheter material and diameter may affect the method execution. In larger carp, slightly stiffer yet conservatively sized catheters improved guidance between pharyngeal teeth and the masticatory plate of os basioccipitalis; conversely, using a smaller than expected diameter often eased passage without reducing recovery. When a very soft catheter could not be guided between the teeth, the attempt was aborted and repeated with a slightly stiffer catheter of the same or next gauge. This aligns with Faina's guidance that very large catheters (>10 mm) are unnecessary even in big carp and with method syntheses emphasising size‐appropriate tooling and explicit reporting of device dimensions and handling safeguards (Chipps & Garvey, 2007; Hyslop, 1980; Kamler & Pope, 2001; Manko, 2016). Our experience supports that emphasis: clear specifications for anaesthesia stage, wetted surfaces, catheter path and insertion limits, infusion rate and recovery checks reduce variability across operators and dates.

Positioning syringe‐based flushing within the broader method toolbox is also important. For gastric species, selective retention can bias diet inferences and pump‐driven systems may be preferable in some settings (Amundsen & Sánchez‐Hernández, 2019). In agastric carp, the simple gut and absence of a pyloric sphincter favour complete evacuations when flow and placement are controlled, making it a suitable non‐lethal option for diet sampling under field conditions. Finally, to aid synthesis across studies, we recommend reporting species and size range, catheter type and diameter, insertion limits, total water passed to clear effluent, welfare safeguards and any season‐specific adjustments. These details enhance reproducibility and allow meaningful comparison of diet datasets generated by non‐lethal methods.

## 5 CONCLUSION

Season‐long syringe‐based gut flushing in pond‐reared carp did not reduce survival or growth relative to handled‐only fish, and a mid‐season difference in July (handled‐only heavier) was transient and absent at harvest. Flushing effort scaled predictably with fish size and season (~20 mL in small fish to >90 mL in large fish, occasional outliers to ~120 mL, highest in late summer), providing simple planning rules for catheter sets and water provisioning. Using rather smaller, rounded, lubricated catheters and guiding them between the pharyngeal teeth (advance ≤2 cm beyond the teeth) yielded clear effluent with low injury risk. Our study provides simple planning rules and confirmed that routine, non‐lethal diet sampling is feasible in agastric cyprinids if catheter size and flushing volume are matched to fish size and season, and basic welfare measures are applied consistently.

## AUTHOR CONTRIBUTIONS

**Lenka Kajgrova:** Conceptualization, data curation, investigation, methodology, writing – original draft. **Petr Blabolil:** Conceptualization, formal analysis, investigation, visualisation, writing – review and editing. **Vladislav Draštík:** Investigation, writing – review and editing. **Luboš Kočvara:** Investigation, writing – review and editing. **Tomáš Jůza:** Investigation, writing – review and editing. **Martin Bláha:** Conceptualization, investigation, methodology, validation, supervison, writing – review and editing.

## Supporting information


**FIGURE S1.** Aerial photo of the experimental pond facility. The ponds used during the experiment are marked with an orange full circle.
**FIGURE S2.** Seasonal dynamics of environmental variables in three ponds (A–C) from March to October. Lines connect monthly observations for each pond. Panels show temperature (°C), oxygen saturation (%), conductivity (μS cm⁻¹), pH and transparency (cm). Colours denote pond identity (A = blue, B = green, C = orange).
**FIGURE S3.** A 150‐mL Janett syringe and a set of catheters were used to test this methodology on common carp. Funnels of various sizes for collecting the intestinal contents of carp.
**FIGURE S4.** Schematic illustration of gut flushing in common carp, showing catheter insertion and collection of the gut contents.
