# Supplementary material for: Validating gut flushing as a non‐lethal sampling technique for diet monitoring in agastric fish
Source: J Fish Biol. 2025 Dec 22;108(4):1192–9. doi: 10.1111/jfb.70301 (PMC13193522; doi:10.1111/jfb.70301)
Supplement: Supplementary file 1 — FIGURE S1. Aerial photo of the experimental pond facility. The ponds used during the experiment are marked with an orange full circle. FIGURE S2. Seasonal dynamics of environmental variables in three ponds (A–C) from March to October. Lines connect monthly observations for each pond. Panels show temperature (°C), oxygen saturation (%), conductivity (μS cm⁻¹), pH and transparency (cm). Colours denote pond identity (A = blue, B = green, C = orange). FIGURE S3. A 150‐mL Janett syringe and a set of catheters were used to test this methodology on common carp. Funnels of various sizes for collecting the intestinal contents of carp. FIGURE S4. Schematic illustration of gut flushing in common carp, showing catheter insertion and collection of the gut contents. [file JFB-108-1192-s001.docx]

**Supporting Information**

**Validating gut flushing as a sampling technique for dietary monitoring in agastric fish**

**Kajgrová Lenka ^1,2#^,** **Blabolil Petr ^1,3#^, Draštík Vladislav ^1^, Kočvara Luboš ^1^, Jůza Tomáš ^1^, Bláha Martin ^2^***

^1^ Biology Centre CAS, Institute of Hydrobiology, Na Sádkách 702/7, 370 05 Ceske Budejovice, Czech Republic

^2^ University of South Bohemia in Ceske Budejovice, Faculty of Fisheries and Protection of Waters, Zátiší 728/II, 389 01 Vodňany, Czech Republic

^3^ University of South Bohemia in Ceske Budejovice, Faculty of Science, Branišovská 1760, 370 05 Ceske Budejovice, Czech Republic

**# Authors contributed equally**

*** Corresponding author:** Martin Bláha

University of South Bohemia in České Budějovice, Faculty of Fisheries and Protection of Waters, Zátiší 728/II, 389 01 Vodňany, Czech Republic

E-mail: blaha@frov.jcu.cz


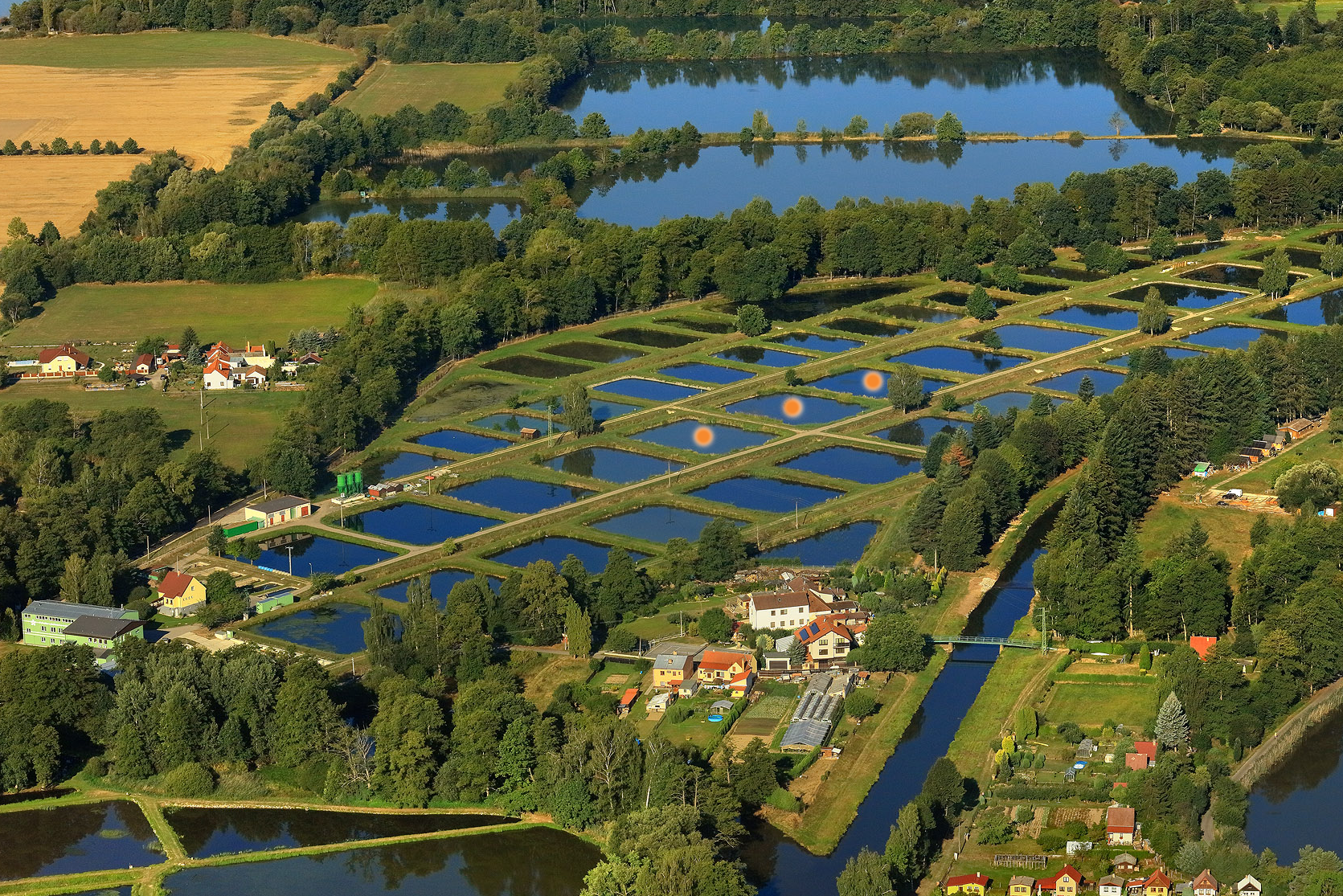


**Figure S1.** Aerial photo of the experimental pond facility. The ponds used during the experiment are marked with an orange full circle.


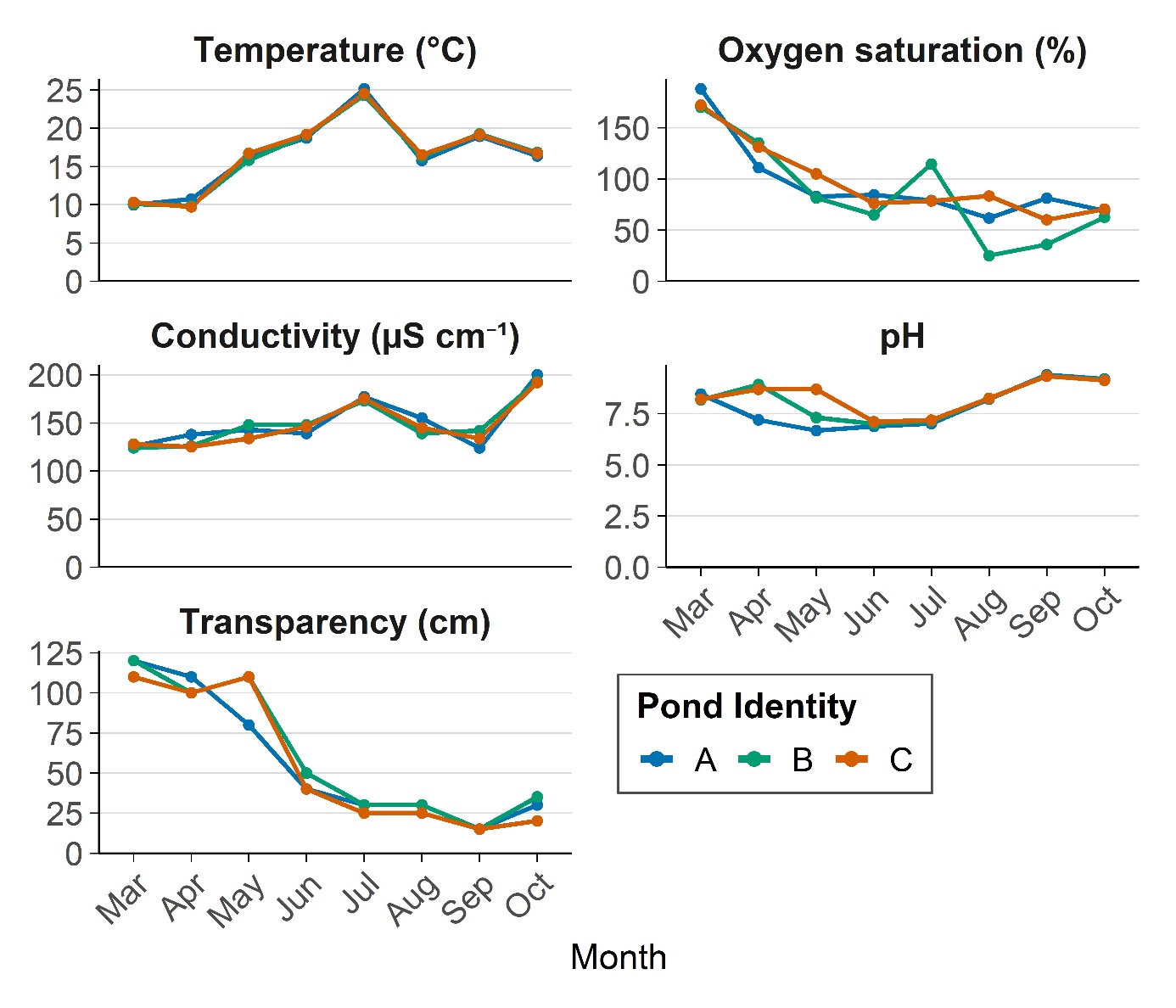


**Figure S2.** Seasonal dynamics of environmental variables in three ponds (A–C) from March to October. Lines connect monthly observations for each pond. Panels show Temperature (°C), Oxygen saturation (%), Conductivity (µS cm⁻¹), pH, and Transparency (cm). Colors denote pond identity (A = blue, B = green, C = orange).


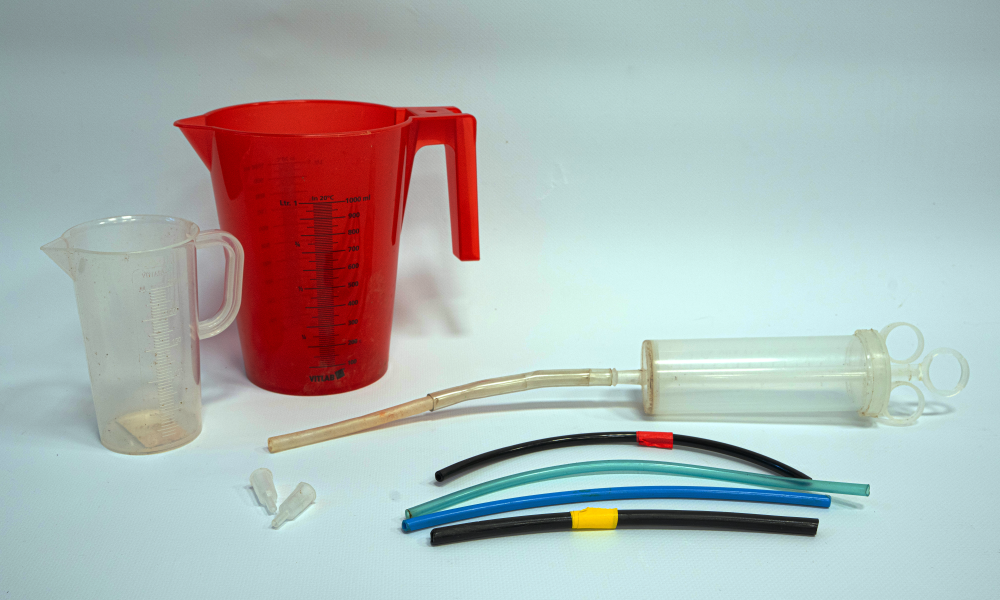


**Figure S3.** A 150 mL Janett syringe and a set of catheters were used to test this methodology on common carp. Funnels of various sizes for collecting the intestinal contents of carp.

**
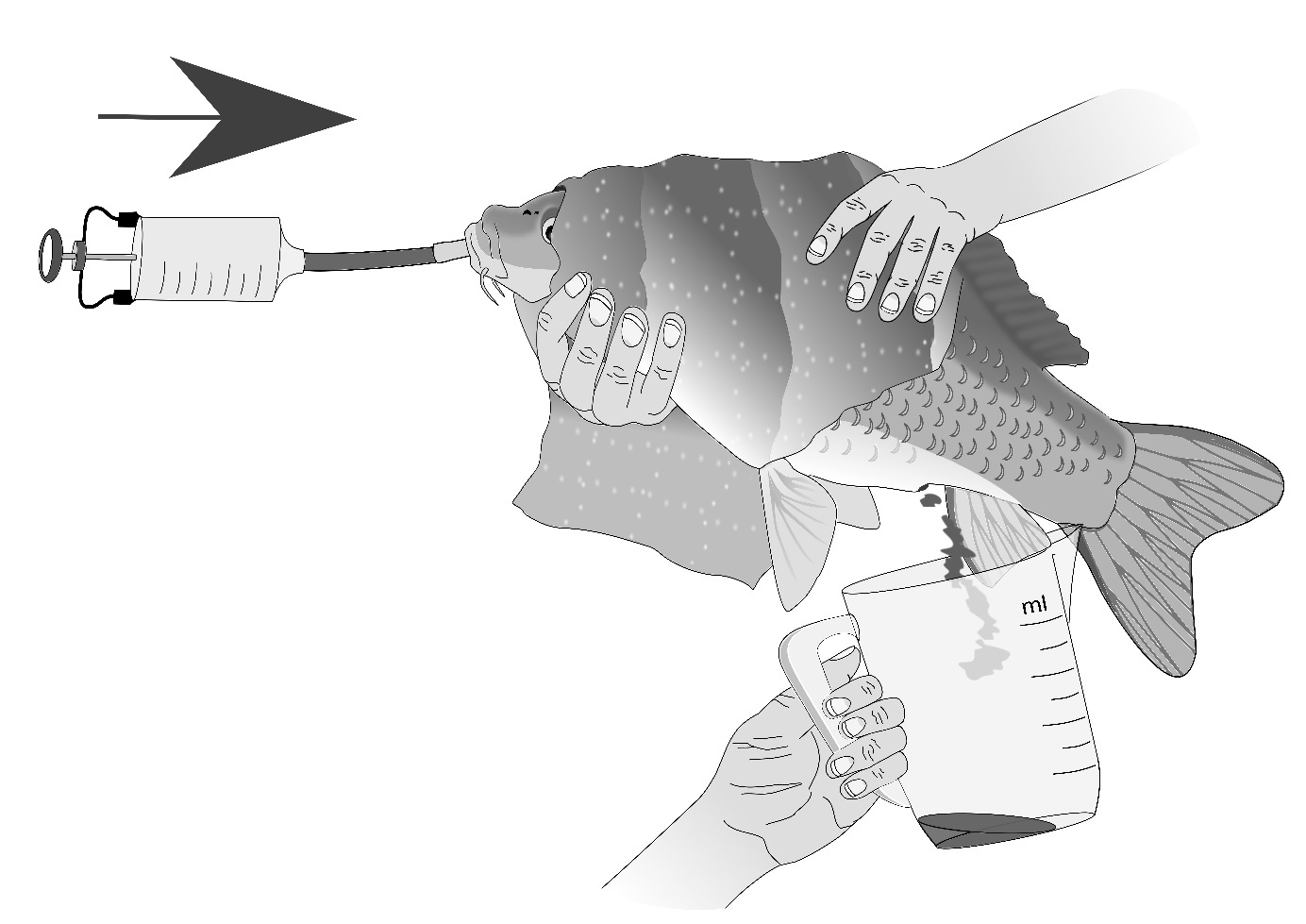
** **Figure S4** Schematic illustration of gut flushing in common carp, showing catheter insertion and collection of the gut contents.
